# Patients’ Perceptions of Barriers and Facilitators to the Adoption of E-Hospitals: Cross-Sectional Study in Western China

**DOI:** 10.2196/17221

**Published:** 2020-06-11

**Authors:** Peiyi Li, Yunmei Luo, Xuexin Yu, Jin Wen, Elizabeth Mason, Weimin Li, Mohammad S Jalali

**Affiliations:** 1 Institute of Hospital Management West China Hospital of Sichuan University Chengdu China; 2 Biomedical Big Data Center West China Hospital of Sichuan University Chengdu China; 3 Massachusetts General Hospital's Institute for Technology Assessment Harvard Medical School Boston, MA United States; 4 Department of Respiratory Medicine West China Hospital of Sichuan University Chengdu China; 5 MIT Sloan School of Management Massachusetts Institute of Technology Cambridge, MA United States

**Keywords:** innovation adoption, e-hospital, internet hospital, eHealth, barriers, facilitators

## Abstract

**Background:**

As an innovative approach to providing web-based health care services from physical hospitals to patients at a distance, e-hospitals (ie, extended care hospitals through the internet) have been extensively developed in China. This closed health care delivery chain was developed by combining e-hospitals with physical hospitals; treatment begins with web-based consultation and registration, and then, patients are diagnosed and treated in a physical hospital. This approach is promising in its ability to improve accessibility, efficiency, and quality of health care. However, there is limited research on end users’ acceptance of e-hospitals and the effectiveness of strategies aimed to prompt the adoption of e-hospitals in China.

**Objective:**

This study aimed to provide insights regarding the adoption of e-hospitals by investigating patients’ willingness to use e-hospitals and analyzing the barriers and facilitators to the adoption of this technology.

**Methods:**

We used a pretested self-administered questionnaire and performed a cross-sectional analysis in 1032 patients across three hierarchical hospitals in West China from June to August 2019. Patients’ sociodemographic characteristics, medical history, current disease status, proficiency with electronic devices, previous experience with web-based health services, willingness to use e-hospitals, and perceived facilitators and barriers were surveyed. Multiple significance tests were employed to examine disparities across four age groups, as well as those between patients who were willing to use e-hospitals and those who were not. Multivariate logistic regression was also performed to identify the potential predictors of willingness to use e-hospitals.

**Results:**

Overall, it was found that 65.6% (677/1032) of participants were willing to use e-hospitals. The significant predictors of willingness to use e-hospitals were employment status (*P*=.02), living with children (*P*<.001), education level (*P*=.046), information technology skills (*P*<.001), and prior experience with web-based health care services (*P*<.001), whereas age, income, medical insurance, and familiarity with e-hospitals were not predictors. Additionally, the prominent facilitators of e-hospitals were convenience (641/677, 94.7%) and accessibility to skilled medical experts (489/677, 72.2%). The most frequently perceived barrier varied among age groups; seniors most often reported their inability to operate technological devices as a barrier (144/166, 86.7%), whereas young participants most often reported that they avoided e-hospital services because they were accustomed to face-to-face consultation (39/52, 75%).

**Conclusions:**

We identified the variables, facilitators, and barriers that play essential roles in the adoption of e-hospitals. Based on our findings, we suggest that efforts to increase the adoption of e-hospitals should focus on making target populations accustomed to web-based health care services while maximizing ease of use and providing assistance for technological inquiries.

## Introduction

The vast majority of high-quality medical resources (eg, well-trained medical workers and advanced medical equipment) are focused in tertiary hospitals in the urban cities of China. This has led to a lack of patients in secondary hospitals and primary health care centers (PHCs), as well as overcrowding in tertiary hospitals. Consequently, patients in tertiary hospitals have long registration and queue times, long waiting times, long dispensary and payment queue times, and short physician visit times, coined as the “three long and one short” condition [1].

In order to reduce the unbalanced distribution of medical resources, China has developed a hierarchical medical treatment system, in which medical institutions of various levels receive and treat patients according to the severity and urgency of their diseases [2,3]. Specifically, tertiary hospitals treat patients with complex and urgent conditions, whereas secondary hospitals treat patients with common diseases. Providers in PHCs have the responsibility of chronic disease management, and they refer patients to specialists or hospitals when necessary [4]. However, despite a great amount of financial investment [5], many PHCs still have poor clinical performance and deficient medical knowledge, as they lack well-trained and qualified physicians [6,7]. Specifically, previous research has found that more than half of the health practitioners in China do not have a bachelor’s degree, and the education level is particularly low for providers in rural areas [8]. Therefore, crowds of patients continue to travel to overfilled top-level hospitals in pursuit of quality care at the cost of escalating health care expenditure and time [9].

Major developments in information and communication technology (ICT) and increased prevalence of electronic devices have enabled innovations in health delivery in developed countries, such as the United States, the United Kingdom, and Canada, particularly for low-resource and underserved communities [10-13]. The World Health Organization defines electronic health (eHealth) as “the use of ICT to support the delivery of health services and the management of health systems” [14]. Studies on eHealth interventions have found positive effects for various diseases, including symptom reduction, improved health care accessibility, and higher patient and clinician satisfaction [15-18].

Similar benefits have resulted from eHealth efforts in China as well [19]. As a priority eHealth project, e-hospitals were proposed by the National Health Commission of China as an innovative approach to health care service delivery in 2015 [20], and they were expected to alleviate the dilemmas regarding accessibility, cost, and quality [21]. There are two major kinds of e-hospitals. There are e-hospitals that are administrated by physical tertiary hospitals, where patients are able to reach physicians in these tertiary hospitals via the internet and are referred to PHCs or secondary hospitals in the region. Other e-hospitals are established by investment companies and have registered physicians from all over the country [22].

E-hospitals, also known as “extended care hospitals,” take the form of a smartphone app or website, and they represent a new approach to outpatient service delivery through the internet [23]. E-hospitals have strengthened the communication pathway between health specialists and patients by overcoming time and distance barriers. Through this technology, health care professionals are able to provide high-quality continuous services to their patients, improve disease management, have more frequent follow-ups, answer patient concerns, and receive appointment requests.

To access these health care services, individuals need to register their information, such as name, age, and gender, at their e-hospital of choice. Once registered, users are able to describe their condition via written text, voice message, or video, and upload relevant documents and laboratory images. This information is conveyed to specialized physicians in tertiary hospitals through a chat platform where the patient and physician can have a remote conversation. Depending on the condition, the physician may issue an e-prescription for users to purchase medicines at the linked web-based pharmacy or make an appointment at a nearby PHC or secondary hospital and provide instructions for treatment remotely. Lastly, e-hospitals can provide posttreatment care and monitoring for patients after they are discharged from a physical hospital [24]. E-hospitals make it possible for patients, regardless of their location, to communicate with skilled medical experts, which thereby improves the efficiency and accessibility of quality medical services while saving indirect health costs [23].

Over the past 4 years, 294 e-hospitals have been established in China to help distribute the health services of tertiary hospitals to patients in distant underserved areas [25]. However, this new medical technology has had a lower adoption rate than expected, as it did not appear to rid tertiary hospitals of the inefficiencies described by the “three long and one short” condition [26]. Furthermore, a large number of these e-hospital programs ended in the early stages due to lack of users [27,28].

The implementation cycle plays a critical role in the effective use of this new technology [29]. A primary issue regarding the implementation cycle of e-hospitals is the inadequate understanding of how to best facilitate individuals’ and organizations’ adoption of the technology [30]. Organizations may fail to successfully implement e-hospitals as a result of a lack of readiness [31,32], which is defined as the preparedness of health care users to adopt eHealth for the provision and management of health services [33]. A number of factors may impact user readiness, which thereby affects the success of eHealth programs and whether the desired health outcomes are achieved [34,35]. At the organizational level, factors, such as coordination with concerned stakeholders and proper training, can impact readiness for eHealth implementation [36,37]. Critical readiness-determining factors for health care providers include the intensity of their current workload and the perceived quality of the new technology [38]. Unsurprisingly, patient readiness is also essential to the success of the implementation cycle of eHealth interventions. Relevant research on this topic has been conducted in Western countries, and actions that overcome financial and technical barriers to facilitate successful adoption should be undertaken [39-42]. Even though these patient attitudes toward eHealth projects can to some extent be contextualized to other countries [43], the lack of studies directly assessing the Chinese population leads to ambiguous policy suggestions for health care administrators and managers of e-hospitals in China.

Taking all these factors into account, this study aimed to (1) examine how familiar patients in Western China are with e-hospitals and how willing they are to adopt the technology; (2) investigate the potential factors that influence patient adoption of e-hospitals; and (3) explore various patient perceptions of the advantages and disadvantages of using e-hospitals. We intended for our results to provide evidence-based insights for policy regarding the integration of e-hospitals into China’s health care system and ultimately deepen the adoption of e-hospitals in China.

## Methods

### Setting and Ethical Consent

This cross-sectional study was conducted in three hospitals in Chengdu, the capital of Sichuan province, located in West China, with a population of roughly 83.4 million people [44]. For context, there are more PHCs in Sichuan than any other province in China [45]. In an attempt to mitigate selection bias, the study included patients from all hospital tiers as follows: a tertiary hospital (West China Hospital of Sichuan University), a secondary hospital (First People’s Hospital of Longquan District), and PHCs (Community Health Centers of Chenghua District). Institutional review board approval was obtained from the Research Ethics Committee of the West China Hospital, Sichuan University.

### Study Design and Population

All adult patients (aged 18 years or above) presenting at the aforementioned hospitals from June to September 2019 were asked to complete an anonymous self-administered questionnaire in the physicians’ offices of corresponding departments. No restriction for patients’ diseases or health care status was an approach to remediate selection bias in our study. Respondents were provided with information about the objectives and scope of the survey and were asked, without incentives, to provide consent to participate in the study. Those who agreed to participate were administered the questionnaire by a research investigator, and careful attention was paid that the participants were not in physicians’ offices to avoid potential discomfort. Since acute illnesses are explicitly excluded from e-hospital treatment, patients who went through an emergency procedure were not eligible to participate.

### Instrumentation

Our survey (Multimedia Appendix 1) involved multiple-choice questions divided into the following five categories: (1) sociodemographic and disease characteristics; (2) current usage of electronic devices; (3) previous experience with web-based health care services; (4) willingness to use e-hospitals; and (5) perceived facilitators or barriers regarding e-hospital use. More specifically, depending on the response to part 4, in part 5, participants were asked either what encourages their e-hospital usage or what deters it.

We developed the questionnaire based on a literature review of relevant studies [46,47]. To ensure its validity, we pilot tested the questionnaire using two complementary approaches. First, the questionnaire was evaluated by five experts in the fields of hospital administration, medical informatics, and health care policy, and suggestions were used to increase clarity. Second, we pilot tested the questionnaire with 20 patients and used their feedback to make adjustments to the questionnaire.

### Measures

Our survey consisted of multiple-choice questions regarding sociodemographic, medical, and eHealth usage–related variables. The variables analyzed were age, gender, level of education, employment status (working vs retired), household location (Chengdu vs outside Chengdu), monthly income, and type of medical insurance. Age ranges were selected based on the categorization by the National Bureau of Statistics of China. To clarify, urban employee health insurance is mainly funded by a person’s employer, whereas rural and urban resident health insurances are mainly funded by government subsidies [48]. The response options for monthly income were stratified into four groups (<2000; 2000-5999; 6000-10,000; and >10,000 CNY) [49]. Furthermore, data about whether participants had chronic diseases were recorded to represent the long-term health demand for chronic disease management. Whether a participant had an operation was also recorded to indicate the eventual need for postoperative rehabilitation care.

Furthermore, information technology (IT) skills and living with children (yes vs no) were surveyed to measure participants’ technical skills and the potential for technical support from younger generations, respectively. Overall IT proficiency was measured by evaluating responses regarding “number of owned electronic devices,” “capability to connect to Wi-Fi,” and “capability to install apps.” Each category was given a score of 0 to 5, and then, the scores were summed in order to generate an overall IT score. For binary questions, a score of 0 was given to a “no” response and 5 was given to a “yes” response.

Additionally, participants were asked if they had previous experience with web-based health services (yes vs no). Respondents with previous experience were then asked to indicate the extent of their satisfaction on a 5-point Likert scale (5, extremely satisfied; 4, satisfied; 3, neutral; 2, dissatisfied; and 1, extremely dissatisfied). For evaluation of familiarity with e-hospitals, another 5-point Likert scale was used (5, extremely familiar; 4, quite familiar; 3, know a little bit; 2, only heard the term; and 1, never heard the term).

### Data Collection and Entry

Four trained research staff distributed the paper-based questionnaires to participants. They followed a verbal script and were instructed to address any potential doubts related to the topics covered by the questionnaire. To confirm that the questionnaire was completely filled out, researchers reviewed the responses immediately after the participant completed the questionnaire.

Two authors (PL and YL) experienced in data entry independently entered all case record data into EpiData (version 3.1, EpiData Software). The two Excel spreadsheets were then compared and discrepancies were resolved by checking the original questionnaires, eventually reaching a consensus between the two authors.

### Data Analysis

For statistical analysis, categorical variables were expressed as frequencies and percentages, and continuous variables were expressed as mean values with standard deviations. Descriptive analyses, including the chi-square test, Kruskal-Wallis test, Fisher test, *t* test, and variance analysis, were performed according to the data characteristics. Specifically, multiple significance tests were employed to examine whether there existed differences across age groups. Similarly, statistical significance tests were conducted to examine the association between all other variables and the willingness to use e-hospitals.

Furthermore, a multivariate logistic regression analysis with a range of variables was performed to identify potential indicators of patients’ willingness to use e-hospitals. All variables in the descriptive analysis of the willingness to use e-hospitals were included in the multivariate logistic regression model. Variables with a two-tailed **P* value <*.05 were considered statistically significant. Additionally, a chi-square test was employed to analyze age-related variations in perceived facilitators and barriers.

All statistical analyses were performed using SPSS (version 25, IBM Corp).

## Results

### Participants

A total of 1108 patients completed the survey after 43 patients refused to respond. Seventy-six patients were excluded due to incomplete responses. Overall, this study had a high response rate of 89.7% (1032 patients).

### Sociodemographic and Medical History

The descriptive analysis in Table 1 shows that socioeconomic attributes varied across age groups. Among the participants, 44.4% (458/1032) were male. Among those aged 18 to 34 years, the number of female participants (166/260, 63.8%) was nearly double that of male participants (94/260, 36.2%), although there appeared to be slightly more male participants (139/276, 50.4%) than female participants (137/276, 49.6%) among those aged 65 years or older. In addition, younger participants were more educated than older participants (*P*<.001). Specifically, 64.2% (167/260) of participants aged 18 to 34 years had attended college or above, whereas this was only true for 14.1% to 37.0% of participants in the other three age groups.

The proportion of retired individuals was significantly higher among those aged 65 or older (270/276, 97.8%) than among the other age groups (50-64 years: 164/258, 63.6%; 35-49 years: 21/238, 8.8%; 18-34 years: 4/260, 1.5%). In addition, participants’ monthly income appeared to decline with age (*P*<.001).

Moreover, 55.7% (575/1032) of participants reported living outside of Chengdu. Participants aged 35 years or older appeared to be more likely than younger participants to seek health care services in Chengdu despite not living there (18-34 years: 109/260, 41.9%; 35-49 years: 150/238, 63.0%; 50-64 years: 160/258, 62.0%; 65 years or older: 156/276, 56.5%; *P*<.001). Additionally, nearly half of the older participants did not live with children (50-54 years: 142/256, 55.0%; 65 years or older: 128/276, 46.4%; *P*<.001).

Table 1 also shows that medical history varied across age groups. Older participants appeared to be more likely to have chronic diseases; over 64.9% (179/276) of those aged 65 years or older reported having chronic diseases, whereas this was reported by only 8.5% (22/260) of those aged 18 to 34 years (*P*<.001). Nearly half of the participants (500/1032, 48.4%) underwent an inpatient surgery, and of these, the majority were aged 35 to 64 years. Finally, younger participants more frequently had employee and urban resident insurance than rural and other types of insurance, whereas older participants more frequently had rural resident insurance (*P*<.001).

### Current Usage of Electronic Devices and Web-Based Health Care Services

Results in Table 2 show that younger participants were generally more active in web-based activities. Overall, the mean number of electronic devices owned by those aged 18 to 34 years was 2.4 (SD 1.24) and those aged 65 years or older was 0.54 (SD 0.75) (*P*<.001). Meanwhile, 81.9% (226/276) of those aged 65 years or older reported that they were unable to connect their electronic devices to Wi-Fi and 92.4% (255/276) of these participants also indicated that they did not know how to install a new app.

In addition, there was a statistically significant association between previous usage of web-based medical services and age (*P*<.001). Specifically, 90.8% (236/262) of participants aged 18 to 34 years reported that they had at least once received health services over the internet, whereas this was reported by only 15.9% (44/276) of participants aged 65 years or older. Meanwhile, 83.1% (463/557) of current users indicated that they were satisfied with their web-based medical experiences. Lastly, the proportion of individuals who had never heard of e-hospitals was significantly lower among those aged 18 to 34 years (59/260, 22.7%) than among those aged 65 years or older (241/276, 87.3%).

**Table 1:** Sociodemographic and medical history of the study participants.

Characteristic		Total value	Age stratification in years				*P* value	
			18-34	35-49	50-64	≥65		
Sample size, n		1032	260	238	258	276		
**Gender, n (%)**								.002^a^
	Male	458 (44.4)	94 (36.2)	98 (41.2)	127 (49.2)	139 (50.4)		
	Female	574 (55.6)	166 (63.8)	140 (58.8)	131 (50.8)	137 (49.6)		
**Education level, n (%)**								<.001^b^
	Primary school or below	277 (26.8)	6 (2.3)	40 (16.8)	96 (37.2)	135 (48.9)		
	Junior high school	218 (21.1)	32 (12.3)	62 (26.1)	73 (28.3)	51 (18.5)		
	Senior high school	201 (19.5)	55 (21.2)	48 (20.2)	47 (18.2)	51 (18.5)		
	College or above	336 (32.6)	167 (64.2)	88 (36.9)	42 (16.3)	39 (14.1)		
**Employment status, n (%)**								<.001^c^
	Working	573 (55.5)	256 (98.5)	217 (91.2)	94 (36.4)	6 (2.2)		
	Retired	459 (44.5)	4 (1.5)	21 (8.8)	164 (63.6)	270 (97.8)		
**Monthly income (CNY), n (%)**								<.001^b^
	<2000	381 (36.9)	43 (16.5)	60 (25.2)	139 (53.9)	139 (50.4)		
	2000-5999	418 (40.5)	126 (48.5)	108 (45.4)	89 (34.5)	95 (34.4)		
	6000-9999	158 (15.3)	67 (25.8)	41 (17.2)	20 (7.8)	30 (10.9)		
	≥10,000	75 (7.3)	24 (9.2)	29 (12.2)	10 (3.9)	12 (4.3)		
**Home location, n (%)**								<.001^a^
	Chengdu	457 (44.3)	151 (58.1)	88 (37.0)	98 (38.0)	120 (43.5)		
	Outside of Chengdu	575 (55.7)	109 (41.9)	150 (63.0)	160 (62.0)	156 (56.5)		
**Living with children, n (%)**								<.001^a^
	Yes	728 (70.5)	250 (96.2)	214 (89.9)	116 (45.0)	148 (53.6)		
	No	304 (29.5)	10 (3.8)	24 (10.1)	142 (55.0)	128 (46.4)		
**Having a chronic disease, n (%)**								<.001^a^
	Yes	381 (36.9)	22 (8.5)	57 (23.9)	123 (47.7)	179 (64.9)		
	No	651 (63.1)	238 (91.5)	181 (76.1)	135 (52.3)	97 (35.1)		
**Undergone surgery, n (%)**								<.001^a^
	Yes	500 (48.4)	105 (40.4)	135 (56.7)	148 (57.4)	112 (40.6)		
	No	532 (51.6)	155 (59.6)	103 (43.3)	110 (42.6)	164 (59.4)		
**Medical insurance, n (%)**								<.001^c^
	Employee insurance	456 (44.2)	142 (54.6)	113 (47.5)	95 (36.8)	106 (38.4)		
	Urban resident insurance	163 (15.7)	51 (19.6)	35 (14.7)	49 (19.0)	28 (10.1)		
	Rural resident insurance	368 (35.7)	48 (18.5)	80 (33.6)	109 (42.3)	131 (47.5)		
	Others	45 (4.4)	19 (7.3)	10 (4.2)	5 (1.9)	11 (4.0)		

^a^Chi-square test.

^b^Kruskal-Wallis test.

^c^Fisher test.

**Table 2:** Current usage of electronic equipment and web-based health care services among study participants.

Characteristic		Total value	Age stratification in years				*P* value
			18-34	35-49	50-64	≥65	
Sample size–all, n		1032	260	238	258	276	
Number of electronic devices, mean (SD)		1.51 (4.6)	2.4 (1.24)	1.9 (1.22)	1.28 (1.07)	0.54 (0.75)	<.001^a^
**Able to connect to Wi-Fi, n (%)**							<.001^b^
	Yes	671 (65.0)	259 (99.6)	218 (91.6)	144 (55.8)	50 (18.1)	
	No	361 (35.0)	1 (0.4)	20 (8.4)	114 (44.2)	226 (81.9)	
**Able to install apps, n (%)**							<.001^b^
	Yes	558 (54.1)	257 (98.8)	186 (78.2)	94 (36.4)	21 (7.6)	
	No	474 (45.9)	3 (1.2)	52 (21.8)	164 (63.6)	225 (92.4)	
Information technology skills^c^ score, mean (SD)		7.5 (5.5)	12.3 (1.5)	10.4 (3.7)	6.0 (5.2)	1.8 (3.4)	<.001^a^
**Experience of web-based medical services, n (%)**							<.001^d^
	Yes	557 (54.0)	236 (90.8)	171 (71.8)	106 (41.1)	44 (15.9)	
	No	475 (46.0)	24 (9.2)	67 (28.2)	152 (58.9)	232 (84.1)	
**Degree of knowledge about e-hospitals**							<.001^b^
	Very familiar with	37 (3.6)	22 (8.5)	11 (4.6)	4 (1.6)	0 (0)	
	Know a better bit	46 (4.5)	25 (9.6)	16 (6.7)	3 (1.2)	2 (0.7)	
	Know a good bit	146 (14.1)	73 (28.1)	48 (20.2)	17 (6.6)	8 (2.9)	
	Only heard of	206 (20.0)	81 (31.2)	55 (23.1)	45 (17.4)	25 (9.1)	
	Never heard of	597 (57.8)	59 (22.7)	108 (45.4)	189 (73.3)	241 (87.3)	
Sample size–web-based medicine users, n		557	236	171	106	44	
**Satisfaction with the web-based medical experience, n (%)**							.01^b^
	Extremely satisfied	199 (35.7)	80 (33.9)	63 (36.8)	47 (44.3)	9 (20.5)	
	Satisfied	262 (47.1)	116 (49.2)	80 (46.8)	45 (42.5)	21 (47.7)	
	Neutral	80 (14.3)	35 (14.8)	26 (15.2)	10 (9.4)	9 (20.5)	
	Dissatisfied	10 (1.8)	3 (1.3)	1 (0.6)	2 (1.9)	4 (9.0)	
	Extremely dissatisfied	6 (1.1)	2 (0.8)	1 (0.6)	2 (1.9)	1 (2.3)	

^a^Analysis of variance.

^b^Fisher test.

^c^“Information technology skills” was a combined result of the first, second, and third questions in the relevant part.

^d^Chi-square test.

### Willingness to Use E-Hospitals

It was found that 65.6% (677/1032) of participants were willing to use e-hospitals to manage their disease (Table 3). The results suggested that willingness to use e-hospitals was associated with age (*P*=.04), education level (*P*<.001), employment status (*P*<.001), monthly income (*P*<.001), living with children (*P*<.001), medical insurance type (*P*<.001), chronic diseases (*P*<.001*)*, skillful IT operation (*P*<.001), previous experience of web-based health care services (*P*<.001), and familiarity with e-hospitals (*P*<.001) (Table 3).

**Table 3:** Willingness of participants to use e-hospitals.

Characteristic			Total value		Willingness to use e-hospitals				*P* value
					Yes		No		
Sample size, n (%)			1032		677		355		
Age (years), mean (SD)			50.83 (18.1)		46.49 (16.5)		59.12 (18.1)		.04^a^
**Age stratification (years), n (%)**									<.001^b^
	18-34	260 (25.2)		208 (80.0)		52 (20.0)			
	35-50	238 (23.1)		192 (80.7)		46 (19.3)			
	50-64	258 (25.0)		167 (64.7)		91 (35.3)			
	≥65	276 (26.7)		110 (39.9)		166 (60.1)			
**Gender, n (%)**									.84^b^
	Male	458 (44.4)		302 (65.9)		156 (34.1)			
	Female	574 (55.6)		375 (65.3)		199 (34.7)			
**Education level, n (%)**									<.001^b^
	Primary school or below	277 (26.8)		102 (36.8)		175 (63.2)			
	Junior high school	218 (21.1)		151 (69.3)		67 (30.7)			
	Senior high school	201 (19.5)		148 (73.6)		53 (26.4)			
	College or above	336 (32.6)		276 (82.1)		60 (17.9)			
**Employment status, n (%)**									<.001^b^
	Working	573 (55.5)		444 (77.5)		129 (22.5)			
	Retired	459 (44.5)		233 (50.8)		226 (49.2)			
**Monthly income (CNY), n (%)**									<.001^b^
	<2000	381 (36.9)		189 (49.6)		192 (50.4)			
	2000-5999	418 (40.5)		305 (73.0)		113 (27.0)			
	6000-9999	158 (15.3)		121 (76.6)		37 (23.4)			
	≥10,000	75 (7.3)		62 (82.7)		13 (17.3)			
**Home location, n (%)**									.18^b^
	Chengdu	457 (44.3)		310 (67.8)		147 (32.2)			
	Outside of Chengdu	575 (55.7)		367 (63.8)		208 (36.2)			
**Living with children, n (%)**									<.001^b^
	Yes	728 (70.5)		537 (73.8)		191 (26.2)			
	No	304 (29.5)		140 (46.1)		164 (53.9)			
**Medical insurance, n (%)**									<.001^b^
	Employee medical insurance	456 (4.2)		347 (76.1)		109 (23.9)			
	Urban resident medical insurance	163 (15.7)		116 (71.2)		47 (28.8)			
	Rural cooperative medical insurance	368 (35.7)		181 (49.2)		187 (50.8)			
	Others	45 (4.4)		33 (73.3)		12 (26.7)			
**Having a chronic disease, n (%)**									<.001^b^
	Yes	651 (63.1)		466 (71.6)		185 (28.4)			
	No	381 (36.9)		211 (55.4)		170 (44.6)			
**Undergone surgery, n (%)**									.36^b^
	Yes	500 (48.4)		335 (67.0)		165 (33.0)			
	No	532 (51.6)		342 (64.3)		190 (35.7)			
Information technology skills score, mean (SD)			6.0 (4.6)		9.3 (4.8)		4.0 (5.1)		.002^a^
**Experience of web-based medical services, n (%)**									<.001^b^
	Yes	557 (54.0)		474 (85.1)		83 (14.9)			
	No	475 (46.0)		203 (42.7)		272 (57.3)			
**Degree of knowledge about e-hospitals, n (%)**									<.001^c^
	Very familiar with	37 (3.6)		36 (97.3)		1 (2.7)			
	Know a better bit	46 (4.5)		40 (87.0)		6 (13.0)			
	Know a good bit	146 (14.1)		118 (80.8)		28 (19.2)			
	Only heard of	206 (20.0)		167 (81.1)		39 (18.9)			
	Never heard of	597 (57.8)		316 (52.9)		281 (47.1)			

^a^*t* test.

^b^Chi-square test.

^c^Fisher test.

Table 4 presents the results of the multivariate logistic regression analysis of the willingness to use e-hospitals (all variables from Table 3 were included). The results showed that age did not make a difference after controlling for covariates in the model. Similarly, variations in monthly income, medical insurance type, chronic diseases (yes vs no), and familiarity with e-hospitals could be explained by other covariates.

In contrast, employment status, education level, living with children, IT skills, and previous experience with web-based health care were closely associated with the willingness to use e-hospitals (Table 4). Specifically, employed participants were 1.88 times more likely to be willing to use e-hospitals compared to retired participants (95% CI 1.11-3.18) after adjusting for all other covariates in the model. Furthermore, participants with higher education were more likely to be willing to use e-hospitals (junior high school: OR 1.95, 95% CI 1.21-3.15; senior high school: OR 1.84, 95% CI 1.00-3.37; college or above: OR 2.16, 95% CI 1.09-4.28). In addition, participants living with children had a higher likelihood of being willing to use e-hospitals compared to those living without children (OR 1.88, 95% CI 1.34-2.64). Furthermore, participants with higher IT skills had a higher probability of being willing to use e-hospitals (OR 1.11, 95% CI 1.05-1.17). Lastly, participants with previous experience of using web-based medical services were 2.69 times more likely to be willing to use e-hospitals (OR 2.77, 95% CI 1.80-4.26).

**Table 4:** Multivariate logistic regression of the willingness to use e-hospitals.

Independent variable^a^		Coefficient	Wald *χ*^2^	df	OR (95% CI)	*P* value
Constant		0.796	0.419	1	2.22	N/A^b^
Age		−0.003	0.145	1	0.10 (0.98-1.01)	.70
Gender (male vs female)		−0.251	2.417	1	0.78 (0.57-1.07)	.12
**Education level**						
	Primary school or below	N/A	N/A	N/A	Reference	N/A
	Junior high school	0.667	7.407	1	1.95 (1.21-3.15)	.006
	Senior high school	0.609	3.878	1	1.84 (1.00-3.37)	.049
	College or above	0.768	4.822	1	2.16 (1.09-4.28)	.03
Employment status (working vs retired)		0.632	5.545	1	1.88 (1.11-3.18)	.02
**Monthly income (CNY)**						
	<2000	N/A	N/A	N/A	Reference	N/A
	2000-5999	−0.046	0.038	1	0.96 (0.60-1.51)	.85
	6000-9999	−0.177	0.298	1	0.84 (0.44-1.58)	.59
	≥10,000	0.039	0.008	1	1.04 (0.43-2.49)	.93
Living with children (yes vs no)		0.632	13.272	1	1.88 (1.34-2.64)	.001
Home location (Chengdu vs outside Chengdu)		−0.318	3.026	1	0.73 (0.51-1.04)	.08
**Medical insurance**						
	Employee medical insurance	N/A	N/A	N/A	Reference	N/A
	Urban resident medical insurance	0.026	0.011	1	1.03 (0.63-1.67)	.92
	Rural cooperative medical insurance	−0.056	0.047	1	0.95 (0.57-1.57)	.83
	Others	0.024	0.003	1	1.02 (0.73-1.46)	.95
Having a chronic disease (yes vs no)		0.032	0.032	1	1.03 (0.73-1.46)	.86
Undergone surgery (yes vs no)		0.153	0.823	1	1.17 (0.84-1.62)	.36
Information technology skills score		0.102	13.843	1	1.11 (1.05-1.17)	<.001
Experience with web-based medical services (yes vs no)		1.017	21.282	1	2.77 (1.80-4.26)	<.001
**Degree of knowledge about** **e-hospitals**						
	Very familiar with	N/A	N/A	N/A	Reference	N/A
	Know a better bit	−1.680	2.286	1	0.19 (0.02-1.66)	.13
	Know a good bit	−1.960	3.511	1	0.14 (0.02-1.09)	.06
	Only heard of	−1.752	2.831	1	0.17 (0.02-1.34)	.09
	Never heard of	−1.916	3.385	1	0.15 (0.02-1.13)	.07

^a^Specific analysis for the multivariate logistic regression model: 2lnL=1036.027; Hosmer and Lemeshow test: χ^2^=7.029, *P*=.53.

^b^N/A: not applicable.

### Perceived Facilitators for Users and Barriers for Nonusers

**Figure 1:**
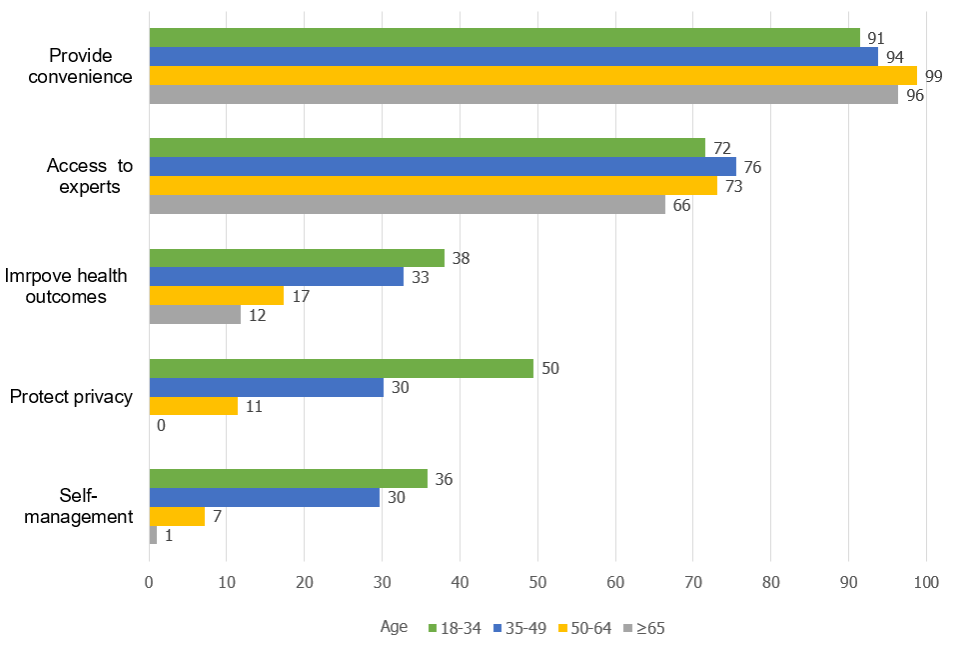
Perceived facilitators for users (%).

The vast majority of participants considered convenience (641/677, 94.7%) to be a major facilitator for e-hospital adoption. The next most popularly agreed upon facilitator was improved access to skilled experts (489/677, 72.2%), followed by improved health outcomes (184/677, 27.2%), privacy protection (180/677, 26.6%), and active participation in disease self-management (144/677, 21.3%). Furthermore, differences in terms of perceived facilitators and barriers appeared to exist across age groups (Figure 1). It was notable that younger participants (aged 18-34 years) showed greater interest in improved health outcomes (79/208, 37.98%; *P*<.001), privacy protection (103/208, 49.5%; *P*<.001), and disease self-management (74/208, 35.8%; *P*<.001) as compared with the other three age groups. In addition, participants aged 50 to 64 years appeared to show greater interest in convenience (165/167, 98.8%; *P*=.04). There was no relevant difference in interest regarding improved accessibility to skilled experts across the age groups (*P*=.32).

**Figure 2:**
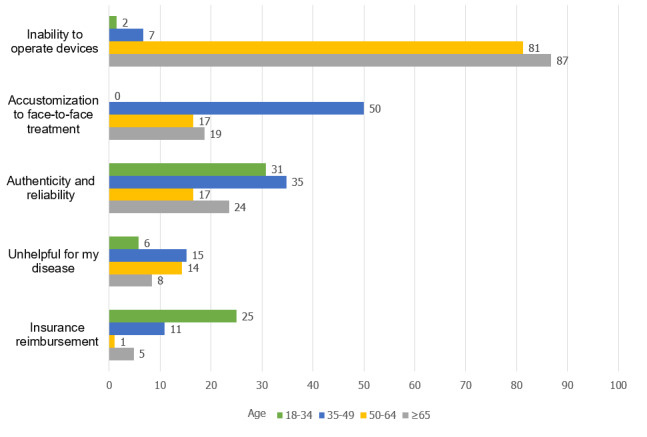
Perceived barriers for non-users (%).

The barriers reported were inability to operate electronic devices (238/355, 67.0%), familiarity with face-to-face health care (108/355, 30.4%), doubts regarding the authenticity and reliability of e-hospitals (86/355, 24.2%), useless perceptions about e-hospitals (37/355, 10.4%), and concerns with insurance reimbursement (27/355, 7.6%). The older groups (aged over 65 years) showed greater concerns with regard to the operation of electronic devices (144/166, 86.7%; *P*<.001) as compared with the other groups (Figure 2). Those aged 35 to 49 years showed more concerns about the authenticity and reliability of e-hospitals (16/46, 34.8%; *P*=.046). Furthermore, young participants (aged 18-34 years) most often reported that they were reluctant to use e-hospitals because they were accustomed to face-to-face treatment (39/52, 75.0%; *P*<.001) and were concerned about insurance reimbursement (13/52, 25.0%; *P*<.001). Lastly, there was no age-related difference when considering “unhelpful for my specific disease” (*P*=.39).

## Discussion

### Study Importance

Research has shown that eHealth programs hold promise for improving health care accessibility and quality [50]; however, this success is contingent on patient acceptance. Therefore, the factors impacting the extent to which people are willing to engage with new eHealth interventions need to be understood in order to facilitate the adoption of these technologies [51,52]. This is the first survey to assess patients’ attitudes, facilitators, and barriers toward e-hospital adoption and to provide related insights for decision makers about the essential factors necessary for successful adoption of e-hospitals in China’s health care system.

### Principal Findings: Part 1

Our results demonstrated that few patients in China know about e-hospitals and even fewer have experience using them. The proportion identified as familiar with e-hospitals (22.2%) in this study is consistent with what has been previously found for this region (18.6%) [53]. Both the prior study and our study indicate that urgent actions should be carried out to ensure broader awareness and familiarity with e-hospitals.

The health care needs and expectations of the older population have reached an unprecedented high in China as a result of reduced mobility and increasing morbidity [54]. In light of this, e-hospitals were theorized as a solution for increasing the efficiency of health care among elderly patients. Although previous research has shown that younger individuals are more likely to use web-based health tools [55,56], our results indicate that this age disparity regarding e-hospital readiness is based on technological proficiency. This may be explained by the pervasiveness of electronic devices in the modern age, which has caused young people to be more technologically proficient as compared with other generations. Nonetheless, this indicates that individuals ordinarily willing to use mobile health, such as elderly patients, may be deterred due to concerns regarding their ability to use technological devices [57,58]. Therefore, even though there is a growing number of e-hospital apps available to support elderly patients in China, their effectivity will likely be minimal unless such apps are designed to be user friendly and accessible for elderly populations.

In addition, our findings revealed that elderly people living with younger generations have higher usage of e-hospitals. This may be related to the fact that seniors living with children are able to get additional instructions and help from relatives. This finding is important, since in recent years, the one-child policy has contributed to reduced family size and an increase in the number of elderly “empty nesters” who are childless or whose children have already left home [59]. As a result, empty nesters’ adoption will be a critical issue given that they are less likely to have access to technical support [60]. Therefore, it would be beneficial to provide solutions, such as offering e-hospital technical services at hospitals and over the internet, to facilitate e-hospital adoption for those who have minimal technological skills [61].

We found that educational disparity is an important determinant of the adoption of e-hospitals, which echoes the findings of previous research [62]. This finding once again underlines the importance of user-friendly interfaces and suggests that app developers should take the needs of people with any education level into account to further minimize the “digital gap” between users and nonusers [63].

Our results also indicated that working participants were more willing to use e-hospitals. This is unsurprising due to the time constraints work imposes on individuals and the convenience of e-hospitals. This is also in accordance with the results of previous studies that found employed individuals to be more interested in eHealth [62]. This indicates that modifying the opening hours of e-hospitals to better accommodate employed patients may play an important role in facilitating adoption.

Furthermore, we found that patients who had previous experiences with web-based medical services expressed positive interest toward e-hospital services. Our findings are in line with the results of previously published surveys, which found correlations between positive attitudes toward web-based health services and interest in eHealth in other contexts [64,65]. However, we found that patients requiring long-term disease management and postoperative rehabilitation did not have much higher willingness to use e-hospitals. This reluctance is congruent with the results of a previous survey that reported low interest toward telemedicine among patients with chronic lung disease [66]. One possibility is that patients with long-standing diseases or poor health conditions are less likely to trust a new less-mature health care delivery model, since they are more aware of the importance of regular thorough self-examination and follow-up. Regardless, these results were not expected, and they contradict one of the aims of e-hospitals, which is to heighten the efficiency and accessibility of chronic disease management and postoperative rehabilitative services. Given that there existed a positive correlation between previous experience with web-based health services and willingness to use e-hospitals, we suggest offering patients who have chronic illnesses or have undergone surgery a cost-free opportunity to get acquainted with e-hospital apps in order to encourage their usage and improve the success of this aim [67].

### Principal Findings: Part 2

Research has shown that receptive patients are most encouraged by the convenience brought about by telemedicine [68], which is consistent with our findings and the current priority of e-hospitals.

However, there was less confidence regarding the “improve health outcomes” facilitator, given that a number of patients reported resistance to e-hospitals due to unassured authenticity and reliability of e-hospitals. Creating an effective e-hospital and integrating it into the system of physical hospitals is a complex endeavor that requires a multipronged strategy that addresses technical and operational issues constrained by local factors. For instance, maintaining the quality of nonprofit medical services is particularly challenging for e-hospitals constructed by for-profit companies. Therefore, establishing a government supervision mechanism [69] and increasing communication among stakeholders may play a key role in assuring the quality of adoption [70]. In addition, evaluation efforts should incorporate robust measures to document the outcomes of e-hospitals and establish a pathway for quick resolution of reported issues.

Given that privacy protection was one of the prominent facilitators for the use of e-hospitals among young individuals, cybersecurity must be prioritized in e-hospitals in order to prevent reluctance to adoption among patients [71,72]. This is substantiated by research [73] reporting that patients were worried about privacy protection when physicians used mobile devices and were particularly concerned with the possibility of individual health data and personal information being exposed. Therefore, we recommend that e-hospitals invest in cybersecurity capability development [74].

Moreover, insurance reimbursement is a key barrier, yet it has not been addressed by any of the 158 e-hospitals in China. In a study conducted in New York, it was reported that nearly half of users stopped using health care apps because of extra costs [46]. To avoid a similar situation, China’s National Healthcare Security Administration should accelerate the pace of integrating e-hospitals into the public medical insurance reimbursement scheme [75].

### Strengths, Limitations, and Future Research

This study has several strengths. First, the sample was relatively large and widely representative, which provided the opportunity for accurate examination of potential variations. Second, participants in the study were very sociodemographically and medically diverse, which allowed for in-depth analysis of variations in attitudes toward e-hospitals among a broad range of patients.

Despite the strengths, this study has several limitations. First, our sampling method was not randomized, and we used convenience sampling, such that participants were patients who happened to visit the hospitals of interest during the period of the survey. Consequently, our results are at risk of having statistical bias. Second, we did not collect text explanations in the survey, which thereby limited the possibility to investigate other facilitators and barriers to the adoption of e-hospitals. Third, as this was a pilot study, our results may not be generalizable to other regions with different sociodemographic characteristics, since we only included patients within one specific context of West China. However, it provided a preliminary analysis, and more research is needed to understand the complexity of the adoption of e-hospitals in China and elsewhere. Additional research will need to be conducted to better understand attitudes toward e-hospitals in other regions. Another limitation is the noninclusion of patients who underwent emergency procedures, and thus, our results do not take into account the possible intricacies of this cohort. Furthermore, this study does not provide insights into the attitudes of those who chose to not participate or complete the questionnaire; therefore, the specific needs and barriers of these users are unknown. We also did not record the demographics of nonresponders. Moreover, we measured connectivity to the internet using Wi-Fi, which is a common approach in the region; however, other forms of connectivity (eg, direct internet access through SIM cards) should be studied. It is hence plausible that there were limitations with the instrument and the collected data.

The integration of e-hospitals into China’s health care system requires an adequate understanding of not only patient attitudes toward acceptance but also the effects of such interventions. Although we identified several themes that can guide the research and technological development of e-hospitals, this study did not elucidate the potential risks of the application of this new technology. To maximize acceptability and usability, future research should focus on user testing with specific e-hospital prototypes. Such user testing should consider including a wider sample of the population, as well as measure the health care outcomes of e-hospital use. In addition, cost-effectiveness analysis is encouraged. Lastly, since the involvement of multiple stakeholders is essential to achieve effective adoption, implementation, and maintenance for systems in practice [76,77], further studies should consider the perspectives of various stakeholders, such as physicians and nurses.

## Appendix

Multimedia Appendix 1 Survey questionnaire for patients’ perceptions of barriers and facilitators to the adoption of e-hospitals.

## Abbreviations

eHealth: electronic health
ICT: information and communication technology
IT: information technology
PHC: primary health care center

## References

[1] Dai J, Wang X, Ayala FJ (2016). Medical Informatics and the "Three Long, One Short" Problem of Large Urban Hospitals in China. JAMA.

[2] Yip WC, Hsiao WC, Chen W, Hu S, Ma J, Maynard A (2012). Early appraisal of China's huge and complex health-care reforms. Lancet.

[3] Li X, Lu J, Hu S, Cheng K, De Maeseneer J, Meng Q, Mossialos E, Xu DR, Yip W, Zhang H, Krumholz HM, Jiang L, Hu S (2017). The primary health-care system in China. Lancet.

[4] (2015). General Office of the State Council.

[5] (2018). State Council People’s Republic of China.

[6] Zeng L, Li Y, Zhang L, Liu G, Zhang Y, Zhen S, Li H, Song X, Duan Y, Yu J, Wang X (2017). Guideline use behaviours and needs of primary care practitioners in China: a cross-sectional survey. BMJ Open.

[7] Du S, Cao Y, Zhou T, Setiawan A, Thandar M, Koy V, Nurumal MSB, Anh H, Kunaviktikul W, Hu Y (2019). The knowledge, ability, and skills of primary health care providers in SEANERN countries: a multi-national cross-sectional study. BMC Health Serv Res.

[8] Wang HH, Wang JJ, Zhou ZH, Wang XW, Xu L (2013). General practice education and training in southern China: recent development and ongoing challenges under the health care reform. Malays Fam Physician.

[9] Yuan B, Balabanova D, Gao J, Tang S, Guo Y (2019). Strengthening public health services to achieve universal health coverage in China. BMJ.

[10] Hesse BW, Greenberg AJ, Rutten LJ (2016). The role of Internet resources in clinical oncology: promises and challenges. Nat Rev Clin Oncol.

[11] Gartner D, Padman R (2017). E-HOSPITAL - A Digital Workbench for Hospital Operations and Services Planning Using Information Technology and Algebraic Languages. Stud Health Technol Inform.

[12] Hartzband P, Groopman J (2010). Untangling the Web--patients, doctors, and the Internet. N Engl J Med.

[13] Agarwal P, Kithulegoda N, Umpierre R, Pawlovich J, Pfeil JN, D'Avila OP, Goncalves M, Harzheim E, Ponka D (2020). Telemedicine in the driver's seat: new role for primary care access in Brazil and Canada: The Besrour Papers: a series on the state of family medicine in Canada and Brazil. Can Fam Physician.

[14] WHO Global Observatory for eHealth (2007). Building foundations for eHealth: progress of Member States: report of the WHO Global Observatory for eHealth.

[15] van den Heuvel JF, Groenhof TK, Veerbeek JH, van Solinge WW, Lely AT, Franx A, Bekker MN (2018). eHealth as the Next-Generation Perinatal Care: An Overview of the Literature. J Med Internet Res.

[16] Jung H, Lee J (2017). The impact of community-based eHealth self-management intervention among elderly living alone with hypertension. J Telemed Telecare.

[17] Das A, Faxvaag A, Svanæs D (2015). The Impact of an eHealth Portal on Health Care Professionals' Interaction with Patients: Qualitative Study. J Med Internet Res.

[18] van der Meij E, Anema JR, Leclercq WK, Bongers MY, Consten EC, Schraffordt Koops SE, van de Ven PM, Terwee CB, van Dongen JM, Schaafsma FG, Meijerink WJ, Bonjer HJ, Huirne JA (2018). Personalised perioperative care by e-health after intermediate-grade abdominal surgery: a multicentre, single-blind, randomised, placebo-controlled trial. Lancet.

[19] Lu C, Hu Y, Xie J, Fu Q, Leigh I, Governor S, Wang G (2018). The Use of Mobile Health Applications to Improve Patient Experience: Cross-Sectional Study in Chinese Public Hospitals. JMIR Mhealth Uhealth.

[20] (2015). State Council People’s Republic of China.

[21] Huckvale K, Wang CJ, Majeed A, Car J (2019). Digital health at fifteen: more human (more needed). BMC Med.

[22] (2018). National Health and Family Planning Commission.

[23] Tu J, Wang C, Wu S (2015). The internet hospital: an emerging innovation in China. Lancet Glob Health.

[24] Xie X, Zhou W, Lin L, Fan S, Lin F, Wang L, Guo T, Ma C, Zhang J, He Y, Chen Y (2017). Internet Hospitals in China: Cross-Sectional Survey. J Med Internet Res.

[25] Qiu Y, Liu Y, Ren W, Qiu Y, Ren J (2018). Internet-Based and Mobile-Based General Practice: Cross-Sectional Survey. J Med Internet Res.

[26] National Bureau of Statistics of China (2018). China Health Statistics Yearbook 2018.

[27] (2019). Tencent.

[28] (2017). Tencent.

[29] Keen P (1991). Shaping the Future. Business Design through Information Technology. Journal of Information Technology.

[30] Kijsanayotin B, Pannarunothai S, Speedie SM (2009). Factors influencing health information technology adoption in Thailand's community health centers: applying the UTAUT model. Int J Med Inform.

[31] Saleh S, Khodor R, Alameddine M, Baroud M (2016). Readiness of healthcare providers for eHealth: the case from primary healthcare centers in Lebanon. BMC Health Serv Res.

[32] Li P, Luo Y, Yu X, Zeng Z, Jin W, Mason E, Li W, Jalali M (2020). Readiness of Healthcare Providers for e-Hospitals: Cross-sectional Analysis in China. SSRN Journal.

[33] Khoja S, Scott RE, Casebeer AL, Mohsin M, Ishaq A, Gilani S (2007). e-Health readiness assessment tools for healthcare institutions in developing countries. Telemed J E Health.

[34] Khoja S, Scott R, Gilani S (2008). E-health readiness assessment: promoting "hope" in the health-care institutions of Pakistan. World Hosp Health Serv.

[35] Kiberu VM, Mars M, Scott RE (2017). Barriers and opportunities to implementation of sustainable e-Health programmes in Uganda: A literature review. Afr J Prim Health Care Fam Med.

[36] Yu P, Li H, Gagnon M (2009). Health IT acceptance factors in long-term care facilities: a cross-sectional survey. Int J Med Inform.

[37] Durrani H, Khoja S, Naseem A, Scott R, Gul A, Jan R (2012). Health needs and eHealth readiness assessment of health care organizations in Kabul and Bamyan, Afghanistan. East Mediterr Health J.

[38] Ruiz Morilla MD, Sans M, Casasa A, Giménez N (2017). Implementing technology in healthcare: insights from physicians. BMC Med Inform Decis Mak.

[39] Gordon NP, Hornbrook MC (2018). Older adults' readiness to engage with eHealth patient education and self-care resources: a cross-sectional survey. BMC Health Serv Res.

[40] Nahm E, Blum K, Scharf B, Friedmann E, Thomas S, Jones D, Gottlieb SS (2008). Exploration of Patients' Readiness for an eHealth Management Program for Chronic Heart Failure. The Journal of Cardiovascular Nursing.

[41] Muigg D, Kastner P, Modre-Osprian R, Haluza D, Duftschmid G (2018). Is Austria Ready for Telemonitoring? A Readiness Assessment Among Doctors and Patients in the Field of Diabetes. Stud Health Technol Inform.

[42] Schwarz F, Ward J, Willcock S (2014). E-Health readiness in outback communities: an exploratory study. Rural Remote Health.

[43] Katz SJ, Moyer CA (2004). The emerging role of online communication between patients and their providers. J Gen Intern Med.

[44] Statistical Bureau Of Sichuan (2018). NBS survey office in Sichuan: Sichuan Statistical Yearbook.

[45] Tao W, Zeng W, Yan L, Yang H, Wen J, Li W (2019). The health service capacity of primary health care in West China: different perspectives of physicians and their patients. BMC Health Serv Res.

[46] Krebs P, Duncan DT (2015). Health App Use Among US Mobile Phone Owners: A National Survey. JMIR Mhealth Uhealth.

[47] Albrecht U, Afshar K, Illiger K, Becker S, Hartz T, Breil B, Wichelhaus D, von Jan U (2017). Expectancy, usage and acceptance by general practitioners and patients: exploratory results from a study in the German outpatient sector. Digit Health.

[48] Meng Q, Mills A, Wang L, Han Q (2019). What can we learn from China's health system reform?. BMJ.

[49] Chengdu Bureau of Statistics (2018). Announcement on the average salary of all employed persons in 2017.

[50] Melchiorre MG, Lamura G, Barbabella F, ICARE4EU Consortium (2018). eHealth for people with multimorbidity: Results from the ICARE4EU project and insights from the "10 e's" by Gunther Eysenbach. PLoS One.

[51] AlBar AM, Hoque MR (2019). Patient Acceptance of e-Health Services in Saudi Arabia: An Integrative Perspective. Telemed J E Health.

[52] Simblett S, Matcham F, Siddi S, Bulgari V, Barattieri di San Pietro C, Hortas López J, Ferrão J, Polhemus A, Haro JM, de Girolamo G, Gamble P, Eriksson H, Hotopf M, Wykes T, RADAR-CNS Consortium (2019). Barriers to and Facilitators of Engagement With mHealth Technology for Remote Measurement and Management of Depression: Qualitative Analysis. JMIR Mhealth Uhealth.

[53] Chen P, Xiao L, Gou Z, Xiang L, Zhang X, Feng P (2017). Telehealth attitudes and use among medical professionals, medical students and patients in China: A cross-sectional survey. Int J Med Inform.

[54] Yip W, Fu H, Chen AT, Zhai T, Jian W, Xu R, Pan J, Hu M, Zhou Z, Chen Q, Mao W, Sun Q, Chen W (2019). 10 years of health-care reform in China: progress and gaps in Universal Health Coverage. The Lancet.

[55] de Veer AJ, Peeters JM, Brabers AE, Schellevis FG, Rademakers JJ, Francke AL (2015). Determinants of the intention to use e-Health by community dwelling older people. BMC Health Serv Res.

[56] Scott Kruse C, Karem P, Shifflett K, Vegi L, Ravi K, Brooks M (2016). Evaluating barriers to adopting telemedicine worldwide: A systematic review. J Telemed Telecare.

[57] Lee J, Nguyen AL, Berg J, Amin A, Bachman M, Guo Y, Evangelista L (2014). Attitudes and preferences on the use of mobile health technology and health games for self-management: interviews with older adults on anticoagulation therapy. JMIR Mhealth Uhealth.

[58] Jiang J, Zhu Q, Zheng Y, Zhu Y, Li Y, Huo Y (2019). Perceptions and Acceptance of mHealth in Patients With Cardiovascular Diseases: A Cross-Sectional Study. JMIR Mhealth Uhealth.

[59] Qian Y, Qin W, Zhou C, Ge D, Zhang L, Sun L (2018). Utilisation willingness for institutional care by the elderly: a comparative study of empty nesters and non-empty nesters in Shandong, China. BMJ Open.

[60] Zhang C, Xue Y, Zhao H, Zheng X, Zhu R, Du Y, Zheng J, Yang T (2019). Prevalence and related influencing factors of depressive symptoms among empty-nest elderly in Shanxi, China. J Affect Disord.

[61] Matthew-Maich N, Harris L, Ploeg J, Markle-Reid M, Valaitis R, Ibrahim S, Gafni A, Isaacs S (2016). Designing, Implementing, and Evaluating Mobile Health Technologies for Managing Chronic Conditions in Older Adults: A Scoping Review. JMIR Mhealth Uhealth.

[62] Sun L, Wang Y, Greene B, Xiao Q, Jiao C, Ji M, Wu Y (2017). Facilitators and barriers to using physical activity smartphone apps among Chinese patients with chronic diseases. BMC Med Inform Decis Mak.

[63] Bailey SC, O'Conor R, Bojarski EA, Mullen R, Patzer RE, Vicencio D, Jacobson KL, Parker RM, Wolf MS (2015). Literacy disparities in patient access and health-related use of Internet and mobile technologies. Health Expect.

[64] Hofstede J, de Bie J, van Wijngaarden B, Heijmans M (2014). Knowledge, use and attitude toward eHealth among patients with chronic lung diseases. Int J Med Inform.

[65] Rising CJ, Bol N, Kreps GL (2015). Age-Related Use and Perceptions of eHealth in Men With Prostate Cancer: A Web-Based Survey. JMIR Cancer.

[66] Duplaga M (2013). The acceptance of e-health solutions among patients with chronic respiratory conditions. Telemed J E Health.

[67] Cranen K, Veld RH, Ijzerman M, Vollenbroek-Hutten M (2011). Change of patients' perceptions of telemedicine after brief use. Telemed J E Health.

[68] Valikodath NG, Leveque TK, Wang SY, Lee PP, Newman-Casey PA, Hansen SO, Woodward MA (2017). Patient Attitudes Toward Telemedicine for Diabetic Retinopathy. Telemed J E Health.

[69] Snowdon DA, Hau R, Leggat SG, Taylor NF (2016). Does clinical supervision of health professionals improve patient safety? A systematic review and meta-analysis. Int J Qual Health Care.

[70] Jalali M, Rahmandad H, Bullock SL, Lee-Kwan SH, Gittelsohn J, Ammerman A (2019). Dynamics of intervention adoption, implementation, and maintenance inside organizations: The case of an obesity prevention initiative. Soc Sci Med.

[71] Jalali MS, Razak S, Gordon W, Perakslis E, Madnick S (2019). Health Care and Cybersecurity: Bibliometric Analysis of the Literature. J Med Internet Res.

[72] Jalali MS, Kaiser JP (2018). Cybersecurity in Hospitals: A Systematic, Organizational Perspective. J Med Internet Res.

[73] Illiger K, Hupka M, von Jan U, Wichelhaus D, Albrecht U (2014). Mobile technologies: expectancy, usage, and acceptance of clinical staff and patients at a university medical center. JMIR Mhealth Uhealth.

[74] Jalali MS, Siegel M, Madnick S (2019). Decision-making and biases in cybersecurity capability development: Evidence from a simulation game experiment. The Journal of Strategic Information Systems.

[75] (2019). National Healthcare Security Administration.

[76] Craven MP, Lang AR, Martin JL (2014). Developing mHealth Apps with Researchers: Multi-Stakeholder Design Considerations. Design, User Experience, and Usability. User Experience Design for Everyday Life Applications and Services.

[77] Jalali M, Rahmandad H, Bullock S, Ammerman A (2017). Dynamics of Implementation and Maintenance of Organizational Health Interventions. Int J Environ Res Public Health.

