# Supplementary material for: Patients’ Perceptions of Barriers and Facilitators to the Adoption of E-Hospitals: Cross-Sectional Study in Western China
Source: J Med Internet Res. 2020 Jun 11;22(6):e17221. doi: 10.2196/17221 (PMC7317627; doi:10.2196/17221)
Supplement: Multimedia Appendix 1 [file jmir_v22i6e17221_app1.pdf]

Appendix 1, Survey Questionnaire, for:
Patients' Perceptions of Barriers and Facilitators to the Adoption of e-Hospitals: Cross-sectional Analysis in China

(Translated from the original version in Chinese)

Please answer the following questions in the spaces below. To select an answer, draw a "√" in the corresponding box.

Socio-demographic and disease characteristics			
Gender	□ Male □ Female	Age (year)	
Education	□ Primary school or less □ Junior high school<br>□ Senior high school □ College or above		
Employment status	□ Retired □ Working		
Home location	□ Chengdu □ Outside Chengdu		
Living status	□ Living without children □ Living with children		
Monthly income (CNY)	□ ＜2,000 □ 2,000—5,999 □ 6,000—10,000 □ ＞10,000		
Are you currently suffering from a chronic* disease(s)?	□ Yes<br>□ No		
Have you recently had a surgical procedure?	□ Yes<br>□ No		
Type of medical insurance	□ Urban employee basic medical insurance<br>□ Urban resident basic medical insurance<br>□ Rural new cooperative medical insurance<br>□ Other		
Usage of electronic devices			
Which of the following electronic devices do you currently own?<br>(select all that apply)	□ Smart phone □ Tablet<br>□ Laptop □ Desktop Computer<br>□ Sports bracelet □ None		
Are you able to connect to Wi-Fi by yourself?	□ Yes □ No		
Are you able to install a new app by yourself?	□ Yes □ No		
Previous experience with online medical services			
Have you ever used an online medical service?	□ Yes □ No		
Describe your experience:	□ Extremely satisfied<br>□ Satisfied<br>□ Neutral<br>□ Dissatisfied<br>□ Extremely dissatisfied		

Willingness to use e-hospitals	
Have you ever heard of e-hospitals?	□ Yes, I am familiar with e-hospitals and pay close attention to their development.<br>□ I have seen documents about e-hospitals.<br>□ I have only heard minimally about e-hospitals, but would be willing to learn more in the future.<br>□ I have heard of e-hospitals, but do not know what they are.<br>□ No, I have never heard of e-hospitals.
Are you willing to use an e-hospital to manage your disease?	□ Yes □ No
Perceived facilitators for users and barriers for non-users	
If you are willing to use e-hospitals, what is your reason?<br>(select all that apply)	□ E-hospitals are a convenient and time-efficient way of accessing healthcare services.<br>□ E-hospitals allow me to consult with medical experts from afar.<br>□ E-hospitals can improve my health outcomes.<br>□ E-hospitals allow for more private consultations and minimize embarrassment, especially for infectious diseases.<br>□ E-hospitals prompt engagement in self-care.
If you are not willing to use e-hospitals, what is your reason?<br>(select all that apply)	□ I am not comfortable using smart devices.<br>□ I am accustomed to a face-to-face consultation with physicians.<br>□ I do not think e-hospital services would be helpful for treating my disease.<br>□ I do not trust the authenticity and reliability of e-hospitals.<br>□ I am worried that I may not be reimbursed by my medical insurance for e-hospital services.

* Note: According to our definition, chronic diseases should fall into the following list: chronic non-specific lung disease (asthma, bronchitis, and pulmonary emphysema), cardiac diseases, atherosclerotic disease, cerebrovascular disease (stroke, excluding transient ischemic attacks), diabetes mellitus, malignant neoplasms, osteoarthritis, and rheumatoid arthritis. All of the above chronic diseases should be diagnosed by physicians.
